# A combination of genome-wide association study and transcriptome analysis in leaf epidermis identifies candidate genes involved in cuticular wax biosynthesis in *Brassica napus*

**DOI:** 10.1186/s12870-020-02675-y

**Published:** 2020-10-06

**Authors:** Shurong Jin, Shuangjuan Zhang, Yuhua Liu, Youwei Jiang, Yanmei Wang, Jiana Li, Yu Ni

**Affiliations:** College of Agronomy and Biotechnology, Academy of Agricultural Sciences, Southwest University, Chongqing, 400716 China

**Keywords:** *Brassica napus* L., Cuticular wax, Genome-wide association study, RNA-seq, Single nucleotide polymorphism

## Abstract

**Background:**

*Brassica napus* L. is one of the most important oil crops in the world. However, climate-change-induced environmental stresses negatively impact on its yield and quality. Cuticular waxes are known to protect plants from various abiotic/biotic stresses. Dissecting the genetic and biochemical basis underlying cuticular waxes is important to breed cultivars with improved stress tolerance.

**Results:**

Here a genome-wide association study (GWAS) of 192 *B. napus* cultivars and inbred lines was used to identify single-nucleotide polymorphisms (SNPs) associated with leaf waxes. A total of 202 SNPs was found to be significantly associated with 31 wax traits including total wax coverage and the amounts of wax classes and wax compounds. Next, epidermal peels from leaves of both high-wax load (HW) and low-wax load (LW) lines were isolated and used to analyze transcript profiles of all GWAS-identified genes. Consequently, 147 SNPs were revealed to have differential expressions between HW and LW lines, among which 344 SNP corresponding genes exhibited up-regulated while 448 exhibited down-regulated expressions in LW when compared to those in HW. According to the gene annotation information, some differentially expressed genes were classified into plant acyl lipid metabolism, including fatty acid-related pathways, wax and cutin biosynthesis pathway and wax secretion. Some genes involved in cell wall formation and stress responses have also been identified.

**Conclusions:**

Combination of GWAS with transcriptomic analysis revealed a number of directly or indirectly wax-related genes and their associated SNPs. These results could provide clues for further validation of SNPs for marker-assisted breeding and provide new insights into the genetic control of wax biosynthesis and improving stress tolerance of *B. napus*.

## Background

*Brassica napus* L. (2n = 38, genome AACC) is an allotetraploid crop evolved from natural hybridization between two diploid progenitor species, *Brassica rapa* (AA, 2n = 20) and *Brassica oleracea* (CC, 2n = 18), followed by chromosome doubling about ~ 7500 years ago [1]. It is a globally important oil crop which contributes edible oil, biofuels, and industrial compounds such as plasticizers and stabilizer for plastics, lubricants, and surfactants [2]. However, unfavorable environmental factors induced by climate changes such as drought and high temperature, severely influence its yields and qualities [3, 4]. For stabilizing or enlarging oilseed supply, it is important to improve stress tolerance of *B. napus*. The aerial parts of plants are covered with cuticular wax, a mixture of hydrophobic compounds. Both increased cuticular wax deposition and improved tolerance to water deficiency stress were observed in some transgenic crops with the overexpression of wax-associated genes [5–8], suggesting that this hydrophobic layer could protect plants from abiotic stresses.

Cuticular waxes are consisted of very long chain fatty acids (VLCFAs) and their derivatives such as alcohols, aldehydes, alkanes, ketones, and wax esters [9, 10]. The biosynthesis of these aliphatic wax components involves C_16_-C_18_ fatty acids synthesis in the plastid, C_16_-C_18_ fatty acid elongation in the endoplasmic reticulum (ER), and subsequent modification via either the decarbonylation or the acyl reduction pathway [11].

Many genes involved in cuticular wax biosynthesis and its regulation have been identified in Arabidopsis. However, the genetic basis of cuticular waxes in *B. napus* has still not been fully understood. The wax load on *B. napus* leaves is considerably higher in comparison to that on Arabidopsis leaves. However, the wax composition patterns are similar in these two plant species, both consisting of fatty acids, aldehydes, alkanes (predominant compounds), primary alcohols, secondary alcohols, ketones, and esters. Characterization of a novel dominant glossy mutant *BnaA.GL* revealed that the suppression of the *CER1* (a putative aldehyde decarbonylase gene involving in the biosynthesis of alkane) and other wax-related genes such as *MAH1* (a midchain alkane hydroxylase gene involving in the biosynthesis of secondary alcohol and ketone) and *WSD1* (a wax ester synthase/acyl-CoA:diacylglycerol acyltransferase gene involving in the biosynthesis of wax ester) drastically altered the wax production via the alkane pathway in *B napus* [12]. The product of the *KCS1* encodes a condensing enzyme KCS1 (3-ketoacyl-CoA synthase 1) which is involved in the critical fatty acid elongation process in wax precursor biosynthesis [13]. Overexpression of *BnKCS1–1*, *BnKCS1–2*, and *BnCER1–2* in *B. napus* promoted cuticular wax production and increases drought tolerance [14]. Overexpression of *BnLAS,* a member of the GRAS family of putative transcriptional regulators, resulted in inhibition of growth, delays in leaf senescence and flowering time, and more epidermal wax deposition on transgenic leaves of Arabidopsis [15]. Overexpression of *BraLTP1* in *B. napus*, a lipid transfer protein gene from *B. rapa*, caused abnormal green coloration, reduced wax deposition, and resulted in leaf water loss [16]. However, these progresses are still not enough to elucidate the molecular mechanisms of cuticular wax production in *B. napus*.

Recently, genome-wide association studies (GWAS) based on high-throughput genotyping technologies become available as a powerful alternative for dissecting the genetic architecture of complex traits in crops [17, 18]. In rapeseed, traits including flowering time [19], seed oil content [20], seed weight and seed quality [21], branch angle [22], harvest index [23], and resistance to *Sclerotinia* [24], have been dissected by GWAS. However, up to date, no genome-wide association mapping of wax traits in rapeseed has been reported.

In a previous study, 517 *B. napus* accessions were used to analyze the leaf wax phenotype, and the heritability of wax compositions suggested that wax variations were mainly driven by genetic factors and were possibly suitable for GWAS [25]. Luo et al. [26] also applied GWAS analysis to identify putative SNPs associated with as many as 50 leaf wax traits in *Camelina.* These researches suggest that GWAS is feasible for detecting genes related to wax compositions in *B. napus*. In addition, cuticular waxes are synthesized in the ER of epidermal cells, and therefore, an aerial epidermis transcriptome might be more efficient in identifying the candidate genes involved in the synthesis of wax and cutin [27].

Here, we quantified the levels of total cuticular waxes, wax classes and wax compounds in leaves of 192 *B. napus* accessions for two years. Then, 31 wax traits were used to perform GWAS to detect genes potentially related to cuticular wax biosynthesis. To further aid identification of wax related genes and explore molecular mechanism of wax biosynthesis, expression profiles of all GWAS-identified genes were determined by transcriptome of the leaf epidermis from high- and low-wax load *B. napus* lines. This study first used the GWAS tool and the epidermis transcriptome to identify candidate genes related to *B. napus* wax traits. Our results provided insight into the genetic regulation of *B. napus* cuticular wax metabolism; therefore, laid a foundation for genetic improvement of *B. napus* stress tolerance by wax modification.

## Results

### Phenotypic variations of leaf cuticular wax

A total of 192 *B. napus* accessions were used to characterize the leaf wax profiles in 2016 and 2017. The cuticular wax profiles in this panel were similar to those reported by Tassone et al. [25] and Holloway et al. [28]. The leaf wax was mainly consisted of long-chain fatty acids, aldehydes, alkanes, primary alcohols (1-alcohols), secondary alcohols (2-alcohols), ketones, and alkyl esters. In total 21 predominant compounds were obtained from seven wax classes, such as C_27_ alkane, C_29_ alkane, C_31_ alkane, and C_29_ 2-alcohol, etc. Total wax coverage, amounts of wax classes, and the amount of each predominant wax compound were assessed as single trait. Additionally, the sum of C_29_ alkane, C_29_ ketone and C_29_ 2-alcohol, the three most abundant compounds from same biosynthesis pathway were also assessed as a single trait (Total C_29_). The sum of products from alkane-forming pathway and the sum of products from alcohol-forming pathway were also characterized as single trait, respectively. A complete list of the 31 wax traits was provided in Table 1. Extensive phenotypic variations of these wax traits were observed in two consecutive years (Table 1; Additional file 1: Fig. S1). The total wax coverage ranged from 7.75 to 53.93 μg·cm^− 2^ in 2016 (with an average of 27.44 μg·cm^− 2^) and from 4.23 to 44.83 μg·cm^− 2^ in 2017 (with an average of 18.65 μg·cm^− 2^).

**Table 1:** Phenotypic variations of leaf cuticular wax in the association panel of *Brassica napus*

Traits	Year	Min (μg·cm^− 2^)	Max (μg·cm^−2^)	Mean ± SD (μg·cm^−2^)	CV (%)
Total wax	2016	7.75	53.93	27.44 ± 7.75	28.24
	2017	4.23	44.83	18.65 ± 5.55	29.76
Acids	2016	0.15	2.08	0.44 ± 0.27	60.19
	2017	0.05	0.96	0.18 ± 0.11	60.01
Aldehydes	2016	0.22	2.16	0.73 ± 0.34	46.63
	2017	0.06	1.75	0.50 ± 0.28	55.29
Alkanes	2016	4.39	31.78	14.32 ± 4.39	30.65
	2017	2.69	31.82	11.71 ± 3.98	34.02
2-Alcolols	2016	1.37	9.34	3.48 ± 1.37	39.51
	2017	0.26	4.59	1.60 ± 0.65	40.49
C_29_ Ketone	2016	1.99	14.70	6.39 ± 1.99	31.08
	2017	0.14	7.93	3.90 ± 1.45	37.27
1-Alcohols	2016	0.35	2.63	1.15 ± 0.45	39.14
	2017	0.05	1.52	0.46 ± 0.27	58.59
Esters	2016	0.16	2.29	0.93 ± 0.46	49.79
	2017	0.08	0.78	0.31 ± 0.11	34.83
C_26_ Acid	2016	0.05	1.25	0.28 ± 0.22	75.91
	2017	0.00	0.83	0.11 ± 0.09	79.28
C_28_ Acid	2016	0.00	0.06	0.02 ± 0.01	83.15
	2017	0.00	0.03	0.01 ± 0.01	90.39
C_26_ Aldehyde	2016	0.01	0.07	0.02 ± 0.01	49.32
	2017	0.00	0.04	0.01 ± 0.01	42.94
C_28_ Aldehyde	2016	0.05	1.21	0.17 ± 0.13	80.62
	2017	0.02	0.86	0.13 ± 0.12	92.38
C_30_ Aldehyde	2016	0.10	0.88	0.42 ± 0.19	45.37
	2017	0.02	0.67	0.28 ± 0.12	42.32
C_25_ Alkane	2016	0.00	0.04	0.01 ± 0.01	70.91
	2017	0.00	0.04	0.01 ± 0.01	90.12
C_26_ Alkane	2016	0.00	0.01	0.01 ± 0.00	43.70
	2017	0.00	0.02	0.00 ± 0.00	68.72
C_27_ Alkane	2016	0.03	0.27	0.11 ± 0.05	43.09
	2017	0.03	0.25	0.10 ± 0.04	42.40
C_28_ Alkane	2016	0.03	0.25	0.09 ± 0.03	36.08
	2017	0.01	0.17	0.06 ± 0.02	29.82
C_29_ Alkane	2016	4.27	30.90	10.89 ± 3.07	28.19
	2017	2.23	25.52	11.30 ± 3.83	33.90
C_30_ Alkane	2016	0.03	0.38	0.11 ± 0.06	54.70
	2017	0.04	0.38	0.13 ± 0.05	33.80
C_31_ Alkane	2016	0.01	0.98	0.17 ± 0.15	87.60
	2017	0.00	5.82	0.36 ± 0.72	200.0
C_29_ 2-Alcohol	2016	1.30	8.68	3.30 ± 1.30	39.31
	2017	0.21	4.03	1. 50 ± 0.65	43.49
C_31_ 2-Alcohol	2016	0.01	0.11	0.04 ± 0.02	52.51
	2017	0.01	0.08	0.02 ± 0.01	56.29
C_26_ 1-Alcohol	2016	0.19	1.74	0.85 ± 0.33	38.85
	2017	0.03	0.91	0.34 ± 0.20	58.09
C_28_ 2-Alcohol	2016	0.03	0.93	0.30 ± 0.13	44.32
	2017	0.00	0.61	0.13 ± 0.10	79.04
C_38_ Ester	2016	0.00	0.09	0.02 ± 0.02	99.82
	2017	0.00	0.08	0.01 ± 0.01	99.42
C_40_ Ester	2016	0.01	0.38	0.15 ± 0.09	59.26
	2017	0.01	0.18	0.06 ± 0.03	42.75
C_42_ Ester	2016	0.13	1.34	0.59 ± 0.31	53.05
	2017	0.03	0.39	0.18 ± 0.06	35.83
C_44_ Ester	2016	0.01	0.50	0.16 ± 0.11	67.07
	2017	0.01	0.18	0.06 ± 0.02	38.36
Total C_29_	2016	6.83	47.31	23.51 ± 6.83	29.04
	2017	3.10	35.06	18.43 ± 4.63	28.17
Alkane Pathway	2016	7.00	48.63	24.19 ± 7.00	28.95
	2017	3.61	41.76	17.19 ± 5.12	29.80
1-Alcohol Pathway	2016	0.51	4.56	2.08 ± 0.70	33.73
	2017	0.24	1.97	0.78 ± 0.32	40.73

Note: Total C_29_, the sum of C_29_ Alkane, C_29_ Ketone and C_29_ 2-Alcohol; Alkane Pathway, the sum of products from alkane-forming pathway; 1-Alcohol Pathway, the sum of products from alcohol-forming pathway

A two-way ANOVA analysis indicated that most wax traits were influenced by genotype (G), year (Y) and their interactions (G × Y) (*P* < 0.001), suggesting the indispensable role of environment on wax synthesis regulation (Table 2). Heritability values ranged from 0.60 (C_28_ acid) to 0.84 (C_27_ alkane) for each independent wax trait (Table 2). Most of the wax traits in *B. napus* showed continuous variations and approximated a normal distribution (Additional file 1: Fig. S1), suggesting that the wax traits were controlled by multiple genes.

**Table 2:** ANOVA analysis of wax traits in the association panel

Trait	Source	SS	df	Mean Square	*P*-value	H^2^
Total wax	G	37,981.09	191	197.82	2.41E-57	0.70
	Y	22,267.34	1	22,267.34	2.80E-93	
	G x Y	29,597.49	191	154.15	1.88E-40	
	Error	30,801.61	772	39.90		
Acids	G	24.44	185	0.13	9.60E-13	0.63
	Y	13.94	1	13.94	9.40E-45	
	G x Y	25.00	185	0.14	1.93E-13	
	Error	45.94	744	0.06		
Aldehydes	G	83.74	191	0.44	5.53E-99	0.69
	Y	3.27	1	3.27	2.58E-14	
	G x Y	72.66	191	0.38	1.51E-85	
	Error	41.92	772	0.05		
Alkanes	G	13,825.27	191	72.01	9.11E-118	0.68
	Y	1215.46	1	1215.46	5.41E-34	
	G x Y	12,841.18	191	66.88	4.86E-110	
	Error	5758.86	772	7.46		
2-Alcohols	G	806.35	191	4.20	4.14E-47	0.65
	Y	762.84	1	762.84	4.74E-119	
	G x Y	789.47	191	4.11	1.04E-45	
	Error	756.20	772	0.98		
C_29_ Ketone	G	2298.71	191	11.97	2.72E-79	0.72
	Y	1551.63	1	1551.63	8.69E-126	
	G x Y	1718.17	191	8.95	7.71E-56	
	Error	1422.16	772	1.84		
1-Alcohols	G	120.84	191	0.63	8.39E-103	0.77
	Y	109.31	1	109.31	2.14E-179	
	G x Y	68.46	191	0.36	6.62E-54	
	Error	58.20	772	0.08		
Esters	G	89.35	191	0.47	2.15E-12	0.66
	Y	112.97	1	112.97	1.12E-86	
	G x Y	76.70	191	0.40	2.90E-08	
	Error	172.07	772	0.22		
C_26_ Acid	G	49.20	191	0.26	2.14E-27	0.66
	Y	7.43	1	7.43	5.83E-20	
	G x Y	45.93	191	0.24	3.83E-24	
	Error	64.88	772	0.08		
C_28_ Acid	G	0.09	191	0.00	4.12E-03	0.60
	Y	0.00	1	0.00	1.28E-03	
	G x Y	0.10	191	0.00	1.83E-04	
	Error	0.28	772	0.00		
C_26_ Aldehyde	G	0.09	191	0.00	1.19E-35	0.66
	Y	0.01	1	0.01	2.83E-19	
	G x Y	0.08	191	0.00	1.67E-31	
	Error	0.10	772	0.00		
C_28_ Aldehyde	G	24.02	191	0.13	1.40E-77	0.66
	Y	0.22	1	0.22	9.19E-04	
	G x Y	23.11	191	0.12	2.93E-74	
	Error	15.16	772	0.02		
C_30_ Aldehyde	G	22.21	191	0.12	9.30E-89	0.72
	Y	4.42	1	4.42	3.55E-53	
	G x Y	15.96	191	0.08	1.01E-60	
	Error	12.37	772	0.02		
C_25_ Alkane	G	0.05	191	0.00	9.51E-69	0.67
	Y	0.01	1	0.01	2.04E-48	
	G x Y	0.05	191	0.00	1.82E-63	
	Error	0.04	772	0.00		
C_26_ Alkane	G	0.15	191	0.00	4.45E-75	0.66
	Y	0.00	1	0.00	2.64E-03	
	G x Y	0.14	191	0.00	8.02E-73	
	Error	0.10	772	0.00		
C_27_ Alkane	G	1.91	191	0.01	1.63E-160	0.84
	Y	0.04	1	0.04	5.81E-15	
	G x Y	0.71	191	0.00	1.86E-61	
	Error	0.55	772	0.00		
C_28_ Alkane	G	0.44	191	0.00	2.96E-87	0.70
	Y	0.16	1	0.16	1.75E-83	
	G x Y	0.36	191	0.00	5.02E-69	
	Error	0.25	772	0.00		
C_29_ Alkane	G	10,644.50	191	55.44	2.43E-108	0.69
	Y	2092.91	1	2092.91	4.11E-62	
	G x Y	9158.35	191	47.70	1.77E-93	
	Error	4853.36	772	6.29		
C_30_ Alkane	G	1.98	191	0.01	4.04E-18	0.67
	Y	0.50	1	0.50	4.05E-26	
	G x Y	1.70	191	0.01	7.19E-13	
	Error	3.23	772	0.00		
C_31_ Alkane	G	922.16	191	4.80	6.50E-73	0.65
	Y	117.85	1	117.85	2.73E-31	
	G x Y	927.06	191	4.83	2.32E-73	
	Error	614.41	772	0.80		
C_29_ 2-Alcohol	G	713.85	191	3.72	6.55E-44	0.65
	Y	724.19	1	724.19	7.97E-121	
	G x Y	708.50	191	3.69	1.98E-43	
	Error	703.01	772	0.91		
C_31_ 2-Alcohol	G	0.63	191	0.00	4.93E-11	0.65
	Y	0.10	1	0.10	6.53E-15	
	G x Y	0.62	191	0.00	1.87E-10	
	Error	1.28	772	0.00		
C_26_ 1-Alcohol	G	65.90	191	0.34	1.52E-92	0.77
	Y	63.95	1	63.95	1.43E-175	
	G x Y	37.33	191	0.19	1.27E-46	
	Error	35.27	772	0.05		
C_28_ 2-Alcohol	G	10.48	191	0.05	1.23E-97	0.75
	Y	6.04	1	6.04	2.29E-129	
	G x Y	6.50	191	0.03	1.29E-56	
	Error	5.32	772	0.01		
C_38_ Ester	G	0.68	191	0.00	1.21E-03	0.61
	Y	0.05	1	0.05	1.14E-05	
	G x Y	0.70	191	0.00	3.81E-04	
	Error	1.95	772	0.00		
C_40_ Ester	G	3.08	191	0.02	1.78E-10	0.65
	Y	2.05	1	2.05	4.47E-49	
	G x Y	2.74	191	0.01	1.32E-07	
	Error	6.33	772	0.01		
C_42_ Ester	G	34.74	191	0.18	2.05E-11	0.66
	Y	49.55	1	49.55	1.10E-92	
	G x Y	30.50	191	0.16	5.09E-08	
	Error	69.13	772	0.09		
C_44_ Ester	G	11.38	191	0.06	1.29E-02	0.62
	Y	3.74	1	3.74	2.01E-18	
	G x Y	10.81	191	0.06	3.97E-02	
	Error	35.79	772	0.05		
Total C_29_	G	26,185.62	191	136.38	1.60E-104	0.69
	Y	12,555.16	1	12,555.16	2.20E-119	
	G x Y	22,094.52	191	115.08	4.14E-88	
	Error	12,396.99	772	16.06		
Alkane Pathway	G	31,529.40	191	164.22	3.32E-57	0.70
	Y	14,543.28	1	14,543.28	2.02E-77	
	G x Y	24,847.46	191	129.41	5.01E-41	
	Error	25,617.74	772	33.18		
1-Alcohol Pathway	G	460.79	191	2.40	1.14E-13	0.71
	Y	706.92	1	706.92	2.30E-103	
	G x Y	299.06	191	1.56	8.35E-04	
	Error	852.10	772	1.10		

Note:G and Y indicate genotype and year, respectively, and G x Y indicate interaction of G and Y. Total C_29_, the sum of C_29_ Alkane, C_29_ Ketone and C_29_ 2-Alcohol; Alkane Pathway, the sum of products from alkane-forming pathway; 1-Alcohol Pathway, the sum of products from alcohol-forming pathway

High correlation coefficients were observed between C_29_ alkane and C_29_ ketone (r = 0.69) and between C_29_ alkane and C_29_ 2-alcohol (r = 0.67), which were produced from alkane branch pathway, and between 1-alcohol and C_38_ ester (r = 0.87), C_40_ Ester (r = 0.93), and C_42_ ester (r = 0.63), which were produced from alcohol branch pathway (Additional file 2: Table S1). High positive correlation coefficient was also found between the products from alkane-forming pathway and alcohol-forming pathway (r = 0.72), indicating that these wax compositions were not independently regulated (Additional file 2: Table S1).

### Population structure and relative kinship of the association panel

**Fig. 1:**
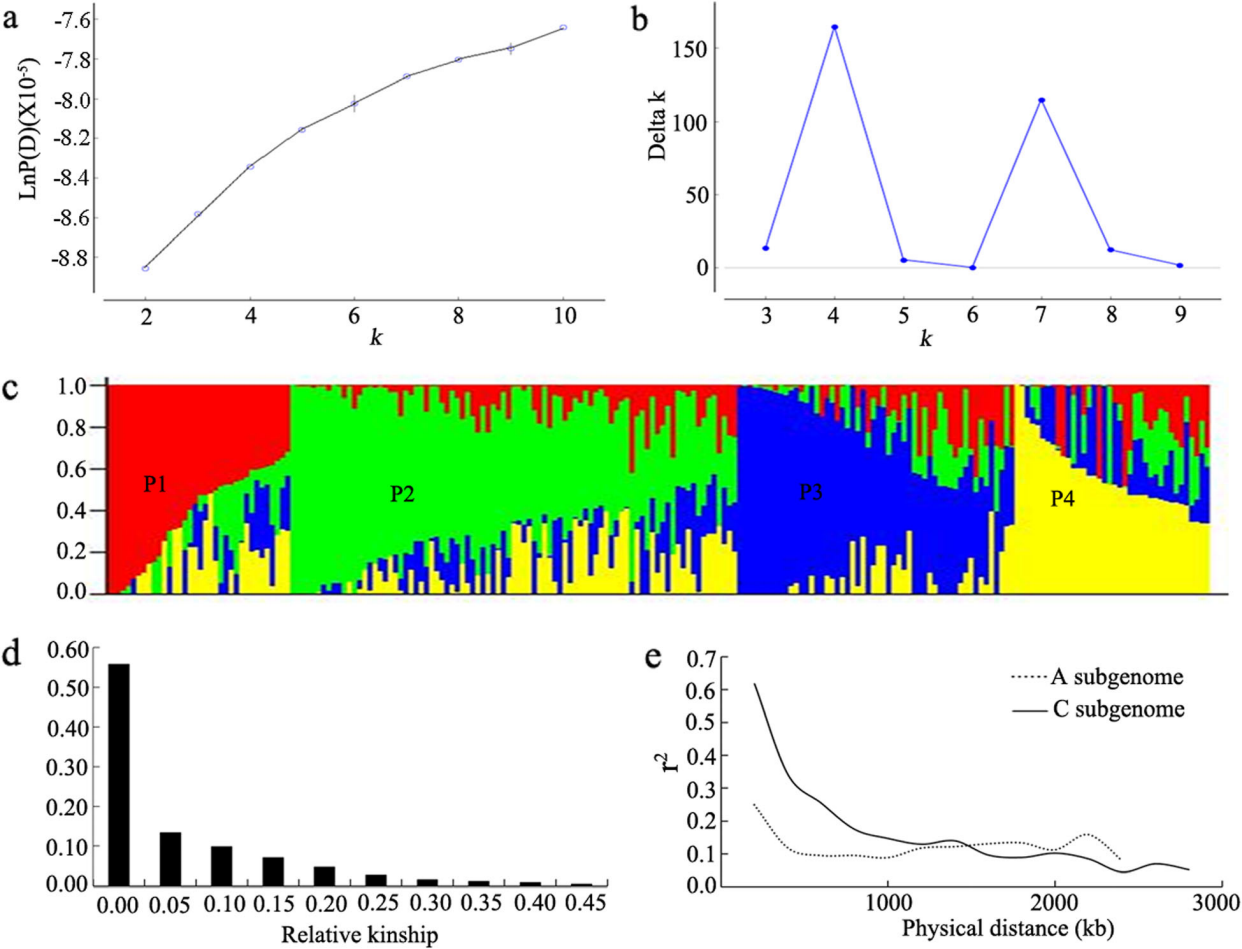
Analysis of linkage disequilibrium decay in two subgenomes and the population structure and relative kinships of 192 rapeseed accessions. **a** Log probability data (LnP(D)) with clusters (K) from 1 to 10 in the STRUCTURE run. **b** ΔK based on the rate of change of LnP(D) between successive K as described by Evanno et al. [28]. **c** Population structure based on K = 4. Red, green, blue and yellow represent sub-population P1, P2, P3, and P4, respectively. Y-axis indicates the composition values belonging to the four sub-populations for a given accession. Each accession is represented by a vertical bar, which is partitioned into colored segments in proportion to the membership in the four sub-populations. **d** Distribution of pairwise kinship in a natural population (192 rapeseed accessions). Only kinship values ranging from 0 to 0.5 are shown. **e** Linkage disequilibrium decay determined by squared correlations of allele frequencies (*r*^*2*^) against distance between polymorphic sites in the A subgenome (the dotted line) and C subgenome (the solid line)

A subset of 4623 SNPs with missing data < 0.2 and MAF > 0.2, which distributed evenly across the entire *B. napus* genome, was selected for population structure and relative kinship analysis (K). Population structure analysis can provide information about the optimal number of subgroups (i.e. the optimal K value) and the proportion of each subgroup in each accession (i.e. Q matrix), which is useful to select the Q matrix corresponding to the optimal K value in the next association analysis, so as to control the false positives caused by the population structure. A clustering inference performed with possible clusters (K) from 1 to 10 showed that the most significant change in likelihood occurred when K increased from 2 to 4 (Fig. 1a), and the highest Δk-value was observed at k = 4 (Fig. 1b). Based on the Δk method described by Evanno et al. [29], the 192 accessions could be divided into four major sub-populations, which were designated as P1, P2, P3, and P4 (Fig. 1c). Most of the spring rapeseed accessions were distributed in P1, while most of the winter accessions were distributed in P2 and P3 (Additional file 3: Table S2).

The analysis of genetic relatedness revealed that 55.8% of the pairwise kinships were equal to 0, and 69% of them ranged from 0 to 0.05 (Fig. 1d), suggesting that most of the accessions in this panel have no or weak kinship, which might be attributed to the broad ranging collections of the genotypes. The results of genetic relatedness analysis would be used in GWAS model as random effect covariate matrix (K matrix) to avoid the false positives in the next association analysis.

The Linkage disequilibrium (LD) decay rate was measured as the chromosomal distance at which the average pairwise correlation coefficient (*r*^*2*^) between all pairs of SNP markers dropped to half of its maximum value. The genome-wide LD decay of A and C subgenome for 192 rapeseed lines were shown in Fig. 1e. The LD of A subgenome decayed faster than that of the C subgenome. The average distance for A subgenome was 500 kb, and for C subgenome was 1600 kb, where *r*^2^ decayed to 0.1.

### Association mapping in *B. napus* for wax traits

**Fig. 2:**
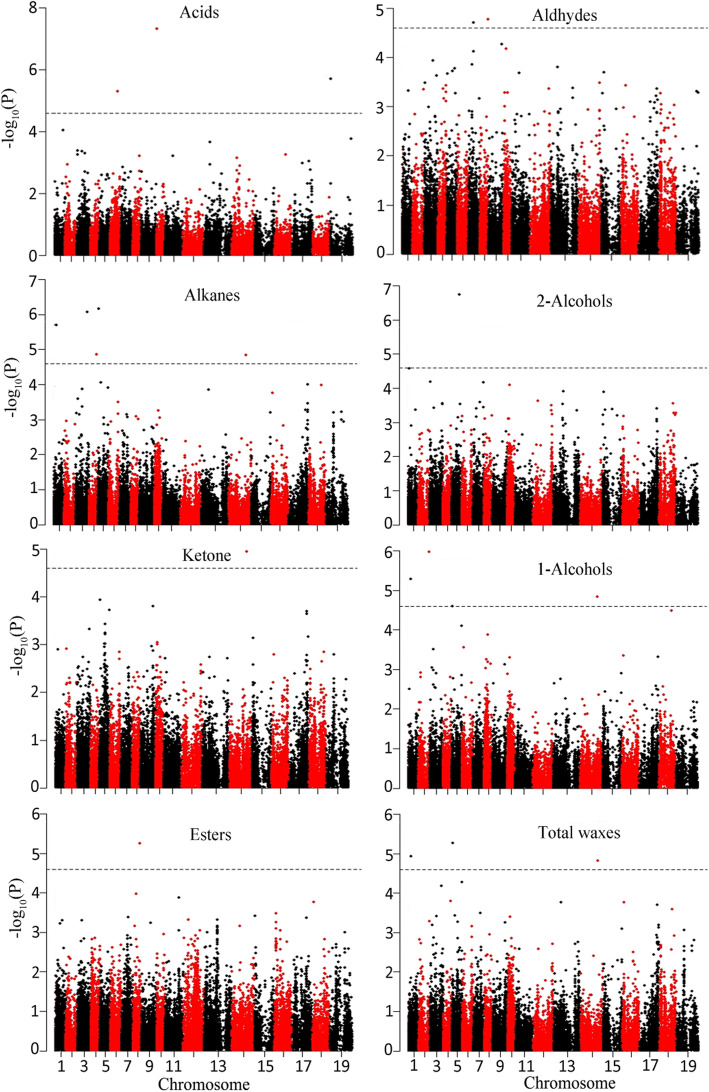
Manhattan plots of GWAS results showing significant SNPs associated with total wax and 7 wax components in rapeseed diversity panel. X-axis shows the distribution of SNPs across 19 chromosomes while Y-axis shows Bonferroni corrections threshold. The black dashed horizontal line depicts the uniform significance threshold [−log_10_(*P*) = 4.5]

To evaluate the effects of population structure (Q, PCA) and kinship (K), six models, including Q, PCA, K, PCA + K, Q + K and naïve (without controlling for Q, PC and K), were separately performed association analysis with the 31 wax traits (Additional file 4: Fig. S2). According to the *P* values from six models, the population structure and kinship can be corrected effectively by the linear mixed model such as PCA + K, Q + K and K when performing GWAS. Eventually, the PCA + K model was selected to perform association mapping for C_28_ acid, C_26_ alkane, C_30_ alkane, C_29_ 2-alcohol, C_29_ ketone, and C_38_ ester, while Q + K model for the remaining 25 wax traits for controlling population structure in GWAS. Thus, a total of 202 significantly associated SNPs for 31 wax traits were identified in a genome-wide scan (*P* < 2.95E-05) (Fig. 2; Additional file 5: Fig. S3; Additional file 6: Table S3). Among these SNPs, 18 were co-associated with multiple wax traits (Additional file 6: Table S3). For example, Bn-A01-p6380934, Bn-A05-p2030789, and Bn-scaff_15798_1-p733219 were simultaneously associated with total wax, alkanes, alkane-forming pathway, and 1-alcohol-forming pathway. The marker Bn-A02-p25285941 was closely related to C_26_ 1-alcohol, total 1-alcohols and 1-alcohol-forming pathway. Bn-A08-p16793918 was closely related to C_38_ ester, C_40_ ester, C_42_ ester, and total esters. Bn-A05-p19622826 was closely related to C_28_ aldehyde, C_29_ 2-alcohol, and total 2-alcohols. According to A- and C- subgenome’s LD decay, genes within ~ 250 kb upstream and downstream to the associated SNPs on A-subgenome and ~ 800 kb on C-subgenome were selected for identification of candidate genes. No SNP was significantly associated with C_30_ aldehyde.

### Genome-wide expression profiles in *B. napus* epidermis based on RNA-seq data

**Fig. 3:**
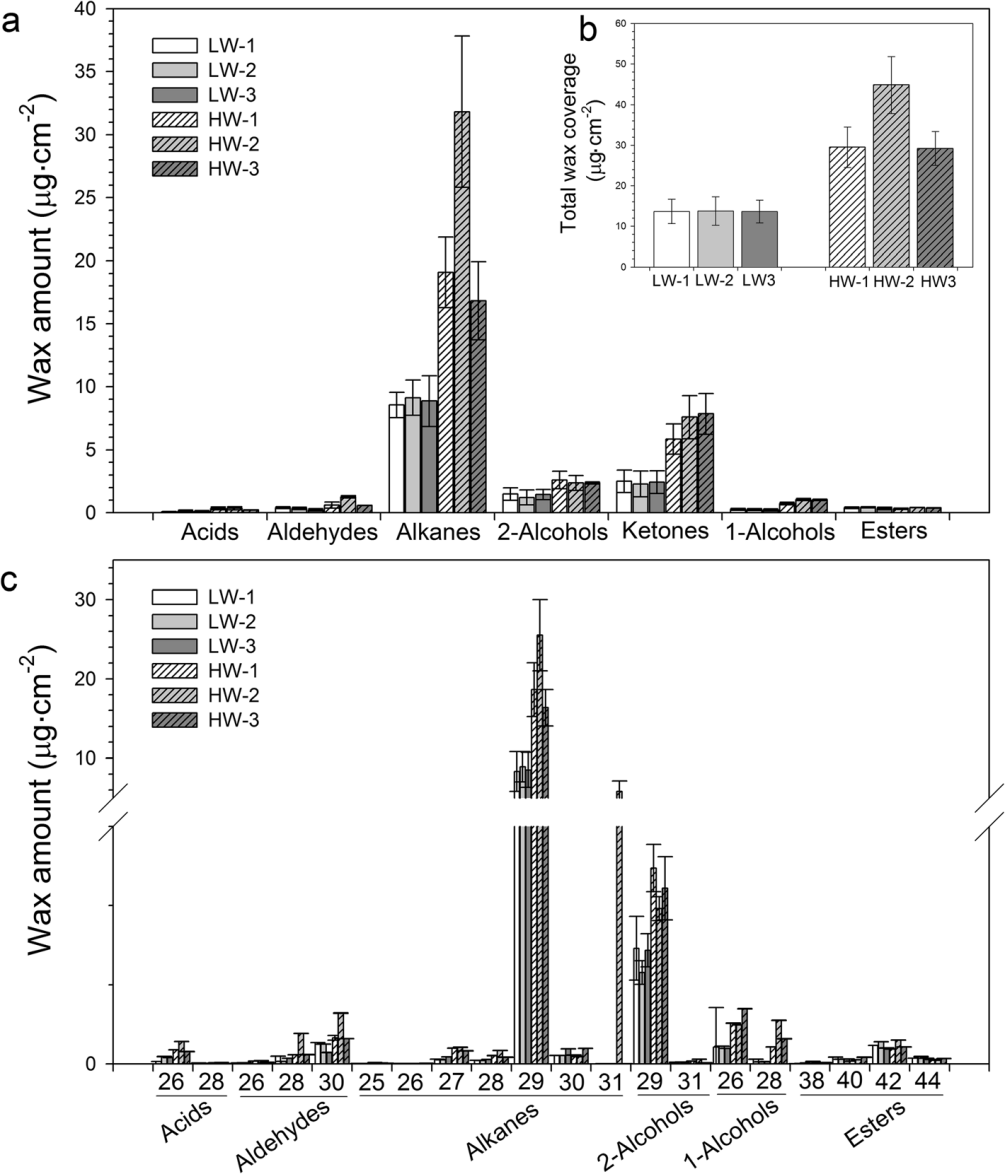
Cuticular wax amounts and composition on rapeseed leaves **a** and wax constituents of fatty acids, aldehydes, alkanes, secondary alcohols, ketone, primary alcohols, and esters on rapeseed leaves **b**. Cuticular waxes were extracted with chloroform and analyzed by GC-FID and GC-MS. The results show averages of three replicates, and error bars indicate ± SD. HW, high wax load rapeseed; LW, low wax load rapeseed

**Fig. 4:**
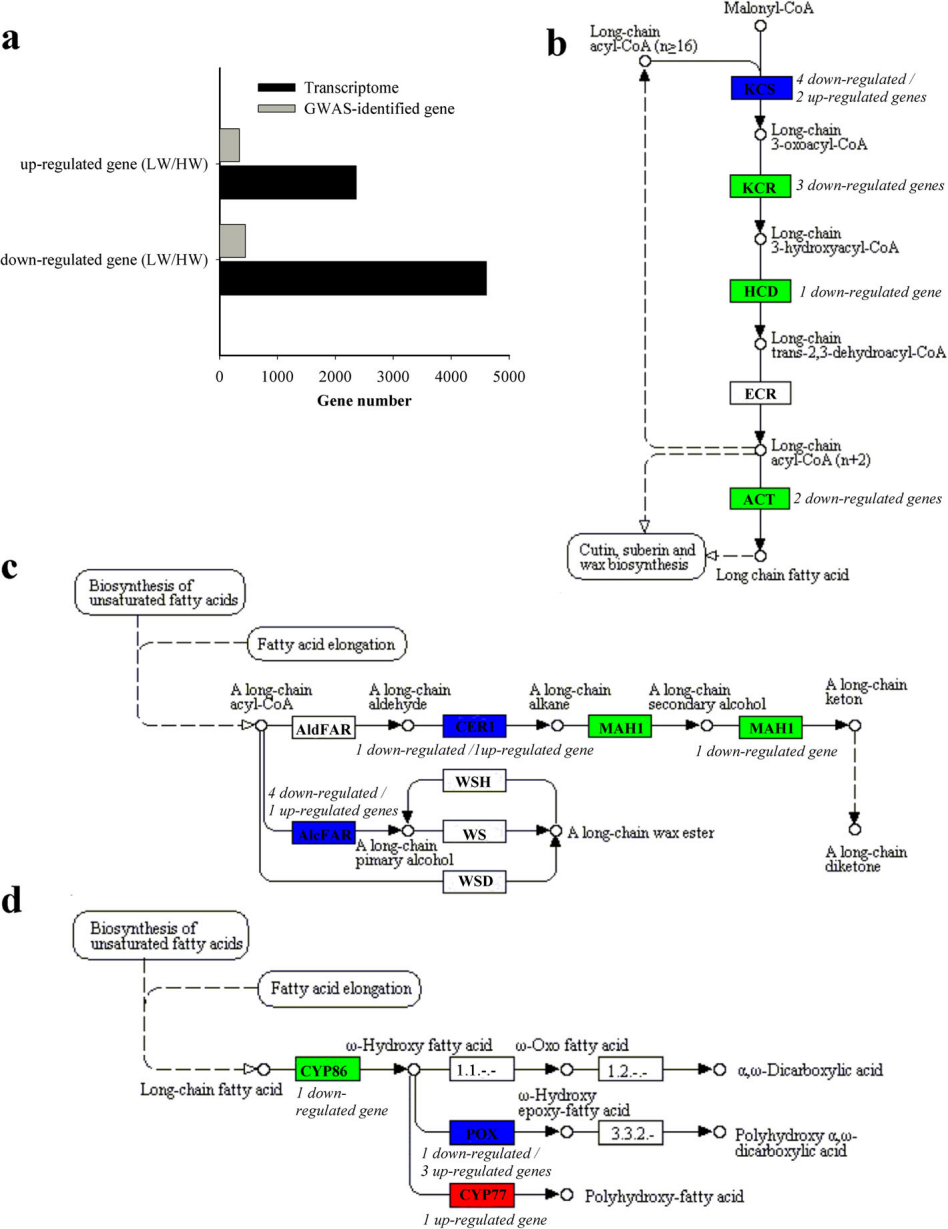
Differentially expressed genes between high-wax load (HW) lines and low-wax load (LW) lines. **a** The number of differentially expressed genes between HW lines and LW lines. **b**, **c** and **d** Annotation map of KEGG pathway of differentially expressed genes involved in fatty acid elongation, wax biosynthesis, and cutin and suberin biosynthesis, respectively. Green represents down-regulation, red represents up-regulation, and blue means up- and down-regulation co-existed in LW lines compared to HW lines. The number of differentially expressed genes encoding a specific enzyme was noted in italics next to the colored box

Next, RNA from epidermis was pooled among high-wax load (HW) lines and low-wax load (LW) lines separately and performed sequencing. The wax load of leaves was 53–70% lower in LW lines when compared to HW lines (Fig. 3b). A reduction in the alkane, 2-alcohol and ketone content was prominent in leaves of LW lines (Fig. 3a and c). By mapping all unique sequences to the *B. napus* genome, a total of 216.49 million mapped reads (258.15 clean reads) were obtained (Additional file 7: Table S4). A high correlation (R^2^ > 0.98) was observed among the three biological replicates, suggesting that the sequencing results were reliable (Additional file 8: Fig. S4). Using software DESeq [30] and FDR < 0.001 and absolute fold change ≥4 as the criteria, a total of 6966 differentially expressed genes (DEGs) between HW and LW lines were detected (Additional file 9: Fig. S5). Among the DEGs, 4608 genes were down-regulated while 2358 genes were up-regulated in LW when compared to HW (Fig. 4a). Since the epidermal samples used in this study possibly contained all epidermal cell types, thus cell type-specific genes, like guard cell-specific genes, could also be included in the DEGs. Among 490 DEGs identified as encoding transcription factors (TFs), 152 were up-regulated in LW when compared to HW, while 338 were down-regulated (Additional file 10: Table S5). Additionally, 20 TFs were only expressed in LW, while 109 TFs only in HW. Among these differentially expressed TFs, 86 belonged to MYB type, 44 belonged to zinc finger type, 40 belonged to AP2/ERF, and the others (Additional file 10: Table S5). About 70% of the MYB, WRKY, ERF and zinc finger type genes were down-regulated in LW, of which 21 MYBs, 7 WRKYs, 3 ERFs and 7 zinc finger types were not expressed in LW. Some orthologs of well-characterized Arabidopsis genes related to wax regulation were identified, such as *MYB16* and *MYB30* (Additional file 10: Table S5). It is reported that the overexpression of *MYB30* in transgenic Arabidopsis plants promoted the production of cuticular wax [31], while MYB16 functioned as a major regulator of cuticle formation in vegetative organs [32]. Our results indicated that MYB family could play a role in the wax regulation of *B. napus*.

### Functional classification of DEGs in the *B. napus* epidermis

To monitor the difference of gene expression pattern between LW and HW lines, Gene Ontology (GO) enrichment analysis was conducted (Additional file 11: Fig. S6). Significantly overrepresented top GO terms of DEGs between HW and LW were enriched in response to stress, cell wall, and transcription factor activity, etc. (Additional file 12: Fig. S7). Among KEGG significantly enriched pathways, 12 DEGs were annotated in fatty acid elongation (ko00062) (Fig. 4b) and 14 DEGs were annotated in wax, and cutin and suberin biosynthesis (ko00073) (Fig. 4 c and d). In the most cases, more than one DEG was assigned to the same enzyme in KEGG pathway.

### Identification of candidate genes

For decreasing false positive error, the expression profile of candidate gene regions on A- and C- subgenome were determined by transcriptome of leaf epidermis from HW and LW lines. Totally, 792 GWAS-identified genes, which associated 147 GWAS-identified SNPs, were revealed to have differential expression between HW and LW lines, including 344 up-regulated genes and 448 down-regulated genes in LW when compared to those in HW (Fig. 4a; Additional file 6: Table S3). KEGG pathway analysis showed that some differentially expressed GWAS-identified genes enriched in fatty acid elongation, wax biosynthesis, and cutin and suberin biosynthesis pathway (Fig. 4b, c and d). Proposed wax-related genes were listed in Table 3, including some reported *A. thaliana* orthologous genes. For example, *BnaA10g00700D*, *BnaC09g16050D* and *BnaC09g51620D* were annotated as *KCS1*, *CER1* and *MAH1*, which were mainly involved in VLCFAs biosynthesis, alkane biosynthesis, and secondary alcohol and ketone biosynthesis, respectively (Fig. 4b and c; Table 3) [13, 33, 34].

**Table 3:** Proposed most likely genes for wax traits by combined GWAS and RNAseq

SNP	Candidate genes in LD interval (number of DEGs)	Wax-related DEGs*	Log_2_FC^a^	Arabidopsis orthologs**	Gene description
Bn-A01-p6380934	BnaA01g11230D-BnaA01g12160D (6)	BnaA01g11780D BnaA01g11880D BnaA01g12090D BnaA01g12130D	3.95 3.64 2.09 −5.46	AT1G14800 AT4G22070 AT4G22270 AT4G22320	Nucleic acid-binding WRKY31 MEMBRANE RELATED BIGGER1 Golgin family A protein
Bn-A01-p2504370	BnaA01g03740D-BnaA01g36700D (92)	BnaA01g03840D	N	AT4G33030**	UDP-Sulfoquinovose Synthase (SQD1)
Bn-A05-p1299570	BnaA05g02080D-BnaA05g36070D (57)	BnaA05g02270D	6.12	AT2G41540**	NAD-dependent Glycerol-3-Phosphate Dehydrogenase (GPDHc1)
Bn-A05-p2030789	BnaA05g03470D-BnaA05g36470D (47)	BnaA05g03540D BnaA05g03570D BnaA05g03920D BnaA05g04000D	−2.47 − 2.19 − 2.59 −3.42	AT2G43770 AT2G43800 AT2G44220 AT2G44450	WD40 repeat-like superfamily protein FORMIN HOMOLOGY 2 DUF239 BGLU15
Bn-scaff_15798_1-p733219	BnaC04g35190D-BnaC04g36140D (3)	BnaC04g35220D BnaC04g35860D	7.51 −4.97	AT1G13080 AT2G23200	CYP71B2 Protein kinase superfamily protein
Bn-A08-p16793918	BnaA08g17640D-BnaA08g18370D (3)	BnaA08g17780D BnaA08g17990D BnaA08g18220D	−2.83 2.04 −6.32	AT1G29720 AT1G29330 AT1G28670	Leucine-rich repeat transmembrane protein kinase ER RETENTION DEFECTIVE 2 (ERD2) ARAB-1/lipase
Bn-A08-p19244116	BnaA08g22500D- BnaA08g23820D (6)	BnaA08g22650D	−2.35	AT1G18360**	Monoacylglycerol Lipase (MAGL)
Bn-A02-p25285941	BnaA02g32000D-BnaA02g32920D (1)	BnaA02g32450D	2.07	AT5G24570	uncharacterized protein
Bn-A05-p19622826	BnaA05g23200D-BnaA05g24000D (2)	BnaA05g23670D BnaA05g23790D	−2.10 −2.60	AT3G15870** AT3G25110**	Acyl-CoA desaturase-like, FAD5-like Desaturase Acyl-ACP thioesterase A (FatA)
Bn-scaff_16361_1-p1569363	BnaC08g27100D-BnaC08g29310D (5)	BnaC08g27970D BnaC08g28370D BnaC08g28510D	−2.09 −6.31 6.77	AT3G57040 AT3G57430 AT1G43760	response regulator 9 (ARR9) ORGANELLE TRANSCRIPT PROCESSING 84 Unknown
Bn-scaff_18636_1-p11498	BnaA01g13360D-BnaA01g14340D (9)	BnaA01g13470D	−2.11	AT4G23850**	Long-chain acyl-CoA synthetase (LACS4)
Bn-A02-p5516551	BnaA02g05260D-BnaA02g06170D (4)	BnaA02g05700D*	4.14	AT5G22500**	Alcohol-forming fatty acyl reductase (AlcFAR1)
Bn-A02-p7004091	BnaA02g06800D-BnaA02g34890D (42)	BnaA02g15790D BnaA02g07110D BnaA02g07120D BnaA02g27940D	3.02 3.44 3.60 −7.00	AT1G72110** AT5G59310** AT5G59310** AT5G54680	Bifunctional wax ester synthase/DAGAT Lipid transfer protein type 1 (LTP4) Lipid transfer protein type 1 (LTP4) bHLH105
Bn-A05-p4055839	BnaA05g06770D-BnaA05g07650D (3)	BnaA05g06830D* BnaA05g07340D	5.35 −3.75	AT2G37700** AT2G37090	CER1-like 2 IRX9
Bn-scaff_20270_1-p1172081	BnaA05g30910D-BnaA05g31670D (1)	BnaA05g31340D	−3.86	AT3G05970**	Long-chain acyl-CoA synthetase (LACS6)
Bn-A05-p23445454	BnaA05g31560D-BnaA05g32450D (10)	BnaA05g32280D BnaA05g32300D BnaA05g32390D BnaA05g32430D BnaA05g32400D BnaA05g32440D	N N N N −8.36 −9.72	AT3G02990 AT3G03050 AT3G03150 AT3G03200 AT3G03160 AT3G03210	HSFA1E CSLD3 Unknown NAC045 Unknown AXY9
Bn-A07-p6765464	BnaA07g08250D-BnaA07g08960D (7)	BnaA07g08340D BnaA07g08720D	5.12 4.39	AT3G25110** AT1G27950**	Acyl-ACP thioesterase A (FatA) Lipid transfer protein type 5 (LTPG1)
Bn-A08-p14906652	BnaA08g13890D-BnaA08g16770D (19)	BnaA08g16470D	I	AT1G47620**	Midchain Alkane Hydroxylase (CYP96A8)
Bn-A09-p30763709	BnaA09g40180D-BnaA09g41120D (4)	BnaA09g40250D BnaA09g40500D	N −4.15	AT5G58470 AT2G26420**	TAF15b Phosphatidylinositol-Phosphate Kinase type IB
Bn-A09-p32945202	BnaA09g44310D- BnaA09g45200D (1)	BnaA09g44740D	4.66	AT1G76690**	Oxo-Phytodienoic Acid Reductase
Bn-A10-p4786596	BnaA10g00010D-BnaA10g28040D (50)	BnaA10g00380D*	−2.18	AT1G01600**	Fatty acyl ω-hydroxylase (CYP86A4)
		BnaA10g00700D* BnaA10g02480D* BnaA10g09300D BnaA10g25660D*	−2.10 − 2.71 N 4.05	AT1G01120** AT1G04220** AT1G08510** AT5G04530**	Ketoacyl-CoA Synthase (KCS1) Ketoacyl-CoA Synthase (KCS2/DAISY) fatty acyl-ACP thioesterases B (FatB) Ketoacyl-CoA Synthase (KCS19)
Bn-A10-p15096523	BnaA10g21560D-BnaA10g24090D (18)	BnaA10g21580D	N	AT1G50410	CHR28
Bn-A03-p12990610	BnaA03g24640D- BnaA03g25500D (12)	BnaA03g24870D BnaA03g24880D	−4.51 −4.95	AT4G11850** AT4G11850**	Phospholipase D &gamma Phospholipase D &gamma
Bn-A03-p18766028	BnaA03g35890D- BnaA03g36690D (2)	BnaA03g36540D	I	AT4G11850**	Phospholipase D &gamma
Bn-A06-p600029	BnaA06g00610D- BnaA06g38780D (57)	BnaA06g00960D BnaA06g08550D BnaA06g08690D	−3.87 4.54 −2.51	AT1G53390** AT1G13560** AT1G13640**	ABC Transporter (WBC25/ABCG24) Diacylglycerol Cholinephosphotransferase (AAPT1) Phosphatidylinositol-4-Kinase &gamma
Bn-A08-p4273091	BnaA08g04100D- BnaA08g04440D (3)	BnaA08g04230D	−3.42	AT1G45201**	Triacylglycerol lipase (TAGL)
Bn-A07-p1657613	BnaA07g01820D- BnaA07g39090D (31)	BnaA07g38540D BnaA07g36830D*	−3.31 −2.25	AT3G45140** AT1G24470**	lipoxygenase 2 (LOX2) Ketoacyl-CoA Reductase (KCR2)
Bn-A04-p14642152	BnaA04g18420D- BnaA04g20360D (3)	BnaA04g19180D BnaA04g19410D* BnaA04g20360D	−2.76 2.61 4.29	AT2G33150** AT2G33380** AT2G34770**	peroxisomal 3-ketoacyl-CoA thiolase (KAT2/FED1) Caleosin (RD20) Fatty Acid 2-hydroxylase (FAH1)
Bn-scaff_17369_1-p884077	BnaC01g15540D- BnaC01g18260D (7)	BnaC01g17900D	−2.23	AT4G25970**	phosphatidylserine decarboxylase 3 (PSD3)
Bn-scaff_15918_1-p77704	BnaC02g38670D-BnaC02g39660D (22)	BnaC02g39310D BnaC02g39360D BnaC02g39080D BnaC02g38980D	N N N N	AT5G28770 AT5G28650 AT5G49460** AT5G49300	bZIP63 WRKY74 ATP citrate lyase B subunit (ACLB-2) GATA16
Bn-scaff_22728_1-p703468	BnaC03g11180D-BnaC03g78270D (16)	BnaC03g12050D BnaC03g72910D BnaC03g73050D	3.37 −5.35 −2.56	AT5G59320** AT4G11850** AT4G11840**	Lipid transfer protein type 1 (LTP3) Phospholipase D &gamma Phospholipase D &gamma
Bn-scaff_16888_1-p1834154	BnaC04g00010D-BnaC04g56960D (14)	BnaC04g11110D* BnaC04g24820D BnaC04g29960D BnaC04g42410D BnaC04g45800D	2.15 N 2.57 −4.59 −3.32	AT2G33380** AT3G56850 AT1G65290** AT2G31380 AT2G38540**	Caleosin (RD20) AREB3 Acyl carrier protein (ACP) salt tolerance homologue Lipid transfer protein type 1 (LTP1)
Bn-scaff_16485_1-p747170	BnaC06g02420D-BnaC06g43990D (4)	BnaC06g14560D BnaC06g28860D BnaC06g43050D BnaC06g43550D	2.21 −2.84 2.21 − 2.20	AT3G53310 AT1G67750 AT1G80080 AT3G25110**	AP2/B3-like TF Pectate lyase ATRLP17 Acyl-ACP thioesterase A (FatA)
Bn-scaff_17526_1-p1726345	BnaC09g00720D-BnaC09g02480D (39) BnaC09g51260D-BnaC09g52120D (2)	BnaC09g01250D BnaC09g01480D BnaC09g01910D BnaC09g01970D BnaC09g51620D*	N N −3.88 −6.39 N	AT3G26935 AT3G27520 AT3G28857 AT3G28920 AT1G57750**	DHHC-type zinc finger family protein Unknown bHLH DNA-binding family protein ZINC FINGER HOMEODOMAIN 9 Midchain alkane hydroxylase (CYP96A15/MAH1)
Bn-scaff_17487_1-p812141	BnaC09g09990D-BnaC09g11020D (19)	BnaC09g10340D BnaC09g10500D BnaC09g10800D	−3.46 −5.41 N	AT2G20300 AT2G20900** AT1G62640**	ALE2 diacylglycerol kinase Ketoacyl-ACP Synthase III (KAS III)
Bn-scaff_20836_1-p125625	BnaC09g15380D-BnaC09g16870D (1)	BnaC09g16050D*	−2.09	AT1G02205**	CER1

^a^ Thresholds for significantly differential expression between high-wax load (HW) and low-wax load (LW) lines were set to false discovery rate (FDR) < 0.001 and fold change ≥4. Positive and negative Log_2_FC values indicate up and down regulation of gene expression in LW when compared to HW. N indicates not expressed in LW and I indicates not expressed in HW

* indicates the genes annotated in Fatty acid elongation pathway, wax, and cutin and suberin biosynthesis pathway in Fig. 4b, c and d

** indicates the genes documented in ARALIP plant acyl lipid metabolism website (http://aralip.plantbiology.msu.edu)

**Fig. 5:**
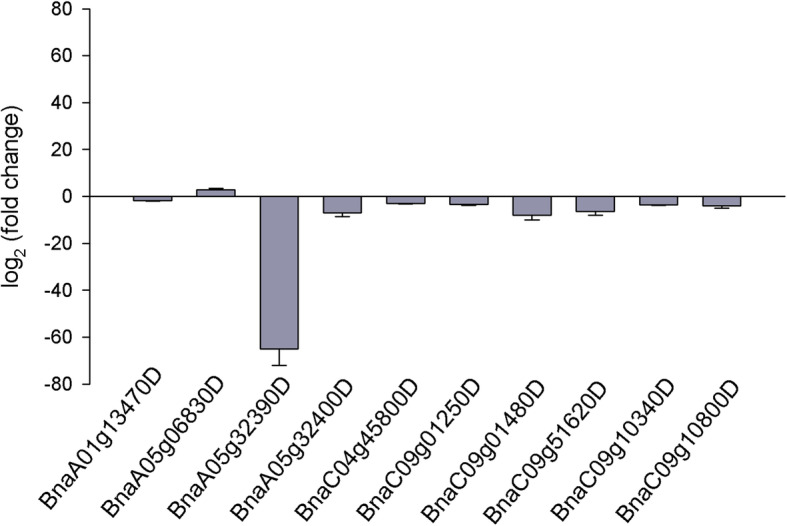
qRT-PCR validation of the expression patterns of 10 genes identified by integrating GWAS with transcriptomic data. The expression in low wax rapeseed relative to high wax rapeseed was calculated as Fold Chang. Values represent the average ± SD of three biological replicates with three technical replicates per sample

To further validate the efficiency of RNA-seq analysis, expression profiles of 10 genes that were commonly identified by GWAS and RNAseq were detected by qRT-PCR. The results showed that the expression changes in these 10 genes from LW and HW were similar to those based on RNAseq analysis (Fig. 5), suggesting the reliability of the RNA-seq data.

## Discussion

*B. napus* is an allotetraploid crop with complex genome structure, which imposes a huge challenge to genome-wide SNP discovery. In the present study, a total of 192 lines were genotyped with the Brassica 60 K SNP array, and the variations in cuticular wax were investigated for two consecutive years. A total of 202 significantly associated SNPs for 31 wax traits were identified in GWAS (Additional file 6: Table S3). These SNP corresponding genes within the LD decay range were selected for identification of the candidates. Considering significant limitations in existing methods for the genome-wide identification of genes, such as false positive and negative results remaining in GWAS [35], we further analyzed the transcriptome of epidermal cells in *B. napus* leaves from HW and LW lines. By integrating GWAS with transcriptomic data, 73% GWAS-identified SNPs, including 792 genes, were revealed to have differential expression between HW and LW lines (Fig. 4a). Although it was impossible to clearly say that these genes were associated with wax traits, they should nevertheless be considered potential wax-related genes.

Based on GO analysis, organic substance metabolic process, cellular metabolic process, primary metabolic process, single-organism cellular process and response to stimulus enriched most of the related genes. A number of genes enriching in cellular process and metabolic process suggested that a complex polygenic network was involved in wax production or deposition in *B. napus*. Cuticular wax is synthesized in the plastids of epidermal cells with the de novo C_16_ and C_18_ fatty acyl-acyl carrier proteins (ACPs) synthesis. *BnaC09g10800D*, the candidate gene in the LD range of the SNP Bn-scaff_17487_1-p812141, encoded ortholog of Arabidopsis *KAS III* [36] and was potentially involved in the de novo fatty acid synthesis. Before being exported to the ER, C_16_ and C_18_ fatty acids were released from ACPs by fatty acyl–ACP thioesterases (FaTA and FaTB) and subsequently esterified to Coenzyme A (CoA) by long-chain acyl-CoA synthetases (LACS) [37]. The acyl-CoA forms of these fatty acids are then elongated to wax precursors of VLCFAs by fatty acid elongase (FAE) complex in the ER. In this study, *BnaA05g23790D*, *BnaC06g43550D* and *BnaA07g08340D*, locating within the LD range of marker Bn-A05-p19622826, Bn-scaff_16485_1-p747170 and Bn-A07-p6765464, separately, encoded FaTA which was potentially involved in the release of C_18_ fatty acid from ACP [38]. *BnaA10g00700D*, *BnaA10g02480D* and *BnaA10g00380D*, the SNP Bn-A10-p4786596 corresponding genes, were annotated as genes encoding KCS1, KCS2 and CYP86A4 in KEGG pathway, which catalyze the biosynthesis of VLCFAs and cutin (Fig. 4b and d) [13, 39, 40]. Overexpression of *BnaA10g00700D/BnKCS1–2* in *B. napus* can promote the production of cuticular wax [14]. *BnaA01g13470D* and *BnaA05g31340D*, two genes in LD to the marker SNP Bn-scaff_18636_1-p11498 and Bn-scaff_20270_1-p1172081, respectively, encoded orthologs of Arabidopsis *LACS4* and *LACS6*, respectively [41, 42], and were potentially involved in the activation from fatty acid to CoA thioesters.

Following elongation, VLC-acyl-CoAs are modified via either the acyl-reduction or the decarbonylation pathway. In the acyl-reduction pathway, fatty acyl-CoA reductase (FAR) catalyzes fatty acyl-CoAs into primary alcohols [10, 11], and wax ester synthase/acyl-CoA: diacylglycerol acyltransferase 1 (WSD1) catalyzes primary alcohols and fatty acid into wax esters [43]. In the decarbonylation pathway, VLC-acyl-CoAs are catalyzed into alkanes by an ER-localized CER1, CER3 and cytochrome B5 complex [33]. It was recently reported that the CER1 homolog, CER1-LIKE1, was also involved in alkane formation [44]. Subsequently, alkanes are oxidized into secondary alcohols and ketones by the midchain alkane hydroxylase 1 (MAH1) [34]. In this study, some DEGs which are homologous to the reported Arabidopsis genes involved in surface lipid biosynthesis were identified within LD blocks. For example, *BnaA02g05700D* located in LD to the marker SNP Bn-A02-p5516551 and encoded ortholog of Arabidopsis *AlcFAR1* (Fig. 4c) [45]. *BnaA02g15790D*, locating in LD to the SNP Bn-A02-p7004091, were annotated as *WSD1*-*like*. *BnaC09g16050D*, the SNP Bn-scaff_20836_1-p125625 corresponding gene, was annotated as Arabidopsis orthologous *CER1* in KEGG pathway (Fig. 4c). Overexpression of *BnaC09g16050D/BnCER1–2* in *B. napus* can promote the biosynthesis of alkane [14]. *BnaA05g06830D*, locating in LD to the SNP Bn-A05-p4055839, were annotated as *CER1*-*like 2* in KEGG pathway. *BnaC09g51620D,* locating in LD to the SNP Bn-scaff_17526_1-p1726345, encoded ortholog of Arabidopsis MAH1 (Fig. 4c) [34]. *BnaC09g10340D*, locating in LD to the SNP Bn-scaff_17487_1-p812141, encoded ortholog of Arabidopsis *ALE2*, which was involved in cuticle development [46].

Wax components synthesized in the ER need to be exported to the extracellular matrix. In the current study, some genes were identified to function in lipid transportation and maintaining ER morphology. For example, *BnaA07g08720D*, locating in LD to the SNP Bn-A07-p6765464, is orthologous to Arabidopsis *LTPG1* which is participated in the output of cuticular wax [47]; *BnaC04g45800D*, locating in LD to the marker Bn-scaff_16888_1-p1834154, is orthologous to *B. rapa LTP1*, which is involved in wax production or deposition as a lipid transfer protein member [48]. *BnaA08g17990D*, locating in LD to Bn-A08-p16793918, is orthologous to *Arabidopsis ERD2* which mediates the transport of soluble ER-localized proteins containing a C-terminal K/HDEL signal [49].

Although the cuticle is usually considered independently from the epidermal cell wall underlying polysaccharide, the cuticle and the cell wall are structurally related (the cuticular layer protrude deeply into cell wall) and have some overlapping functions with each other [50]. Several DEGs related to cell wall were also observed in this study. For example, *BnaA05g32440D* and *BnaA05g32300D*, locating in LD to the SNP Bn-A05-p23445454, encoded orthologs of Arabidopsis *xylan biosynthesis gene AXY9* and *cellulose synthase-like D3* (*CSLD3*), respectively, which were involved in cell wall formation [51, 52].

Some stress-responsive related genes have also been identified, such as *BnaC04g11110D*, which potentially encoded ortholog of *Arabidopsis Responsive to Dehydration gene* (*RD20*), belonging to a member of lipid surface protein family. An increased sensitivity to *Botrytis cinerea* infection and water deficiency was observed in *rd20* mutant [53]. To adapt to dry environment, plants increase leaf cuticular wax deposition to restrict non-stomatal water loss and avoid dehydration [54]. *BnaC02g39310D*, *BnaC02g39360D* and *BnaA02g27940D*, potentially encoding orthologs of Arabidopsis *bZIP63*, *WRKY74* and *bHLH105*/*ILR3* which were involved in modulating responses to starvation, salt tolerance and multiple stress responses [55–57], were also obtained in this study. Plant cuticular wax has been associated with improved plant stress tolerance. In this study, some stress-responsive related genes have been identified by a combined GWAS-RNAseq approach, suggesting that cuticular wax biosynthesis directly or indirectly related with the response to environmental stimulus. However, it remains unknown whether these genes have overlapping effects on wax biosynthesis besides regulating plant responses to stresses. Further studies are required to explore the precise roles of these identified genes.

In this study, some orthologs of well-characterized Arabidopsis wax-related genes could be identified by epidermis transcriptome, such as *BnaC02g37590D* (orthologous to *AtMYB16*) and *BnaC02g05990D* (orthologous to *AtMYB30*) (Additional file 10: Table S5) [31, 32], whereas they couldn’t be identified by GWAS, implying that the role of these genes in wax production might act through variable expressions. Our results showed that combined GWAS and the epidermis transcriptome sequencing analysis could increase the efficiency to detect genes associated with cuticular wax biosynthesis and to prioritize likely candidates, though some wax-related genes that did not vary in gene transcription but had an impact on enzyme function/activity might be overlooked.

## Conclusions

This study first used the GWAS tool and the epidermis transcriptome to identify candidate genes associated with *B. napus* wax traits. A total of 202 SNPs were found to be significantly associated with 31 wax traits. Furthermore, 792 GWAS-identified genes and their associated 147 SNPs were revealed to have differential expressions between HW and LW lines, including 344 up-regulated genes and 448 down-regulated genes in LW when compared to those in HW. These identified SNPs could provide clues for further exploration and validation for marker-assisted breeding, and the proposed wax-related genes could provide new insights into the genetic control of wax metabolism and improving stress tolerance of *B. napus*.

## Methods

### Plant material and experimental design

A total of 192 *B. napus* accessions classified as spring, semi-winter and winter types, were used for an association analysis in this study (Additional file 3: Table S2). All the accessions were provided by the Chongqing Rapeseed Engineering & Technology Research Center, Southwest University, Chongqing, China. Most of these accessions were derived from research institutions in China, with the remaining introduced from Germany, Demark, and Canada. The experiments were conducted at Xiema Experimental Station (29°45′N, 106°22′38E), Beibei, Chongqing, China. The *B. napus* accessions were seeded in randomized complete blocks with three replicates in September, 2016 and 2017. Each accession was planted in two rows, 40 cm between rows, and 20 cm between plants. Routine field management was carried out. Nine weeks after planting (late December, pre-flowering stages for all accessions) uniform and fully expanded leaf samples from all plots were collected for wax trait investigation. The average month temperature in September, October, November and December in 2016 and 2017 were 22.9 and 23.3 °C, 18.9 and 17.4 °C, 12.7 and 13.3 °C, and 9.4 and 8.3 °C, respectively; whereas the month rainfall were 95.7 and 71.9 mm, 96.9 and 178.4 mm, 89.7 and 25.5 mm, and 17.1 and 6.9 mm, respectively.

### Cuticular wax analysis

The cuticular wax composition of leaves from all accessions was determined as described by Wang et al. [8] with some modifications. Three leaves (third leaf from the top) were sampled from three plants in each replicate for each accession. The sampling was finished within three days. Leaf cuticular waxes were extracted in chloroform containing 10 μg tetracosane (Sigma Aldrich, Missoui, USA) as an internal standard. Then the extracts were derivatized with 50 μL N,O–bis (trimethylsilyl) trifluoroacetamide (BSTFA) and 50 μL pyridine. The derivatized extracts were dried and re-dissolved in chloroform for GC-FID analysis. The wax compound was identified and quantified by mass spectrums, internal standard and leaf area. Leaf areas were determined using ImageJ software (http://rsb.info.nih.gov/ij/).

### Statistical analysis

Wax traits was investigated for three replicates in 2016 and 2017 (Additional file 13: Table S6). Each wax trait of each accession was defined as the average of the three replicates in the same year. For each wax trait, best linear unbiased predictors (BLUP) were estimated for each line across the two years based on a linear model using the lme4 package [58]. The BLUP values were used as phenotypes for the association analysis. The broad-sense heritability was calculated as *H*^*2*^ = *δ*_*g*_^*2*^/ (*δ*_*g*_^*2*^ + *δ*_*ge*_^*2*^/*n* + *δ*_*e*_^*2*^/*nr*), where *δ*_*g*_^*2*^ is the genetic variance, *δ*_*ge*_^*2*^ is the interaction variance of the genotype with year, *δ*_*e*_^*2*^ is the error variance, n is the number of years and r is the number of replicates within a year.

### SNP genotyping, filtering and in silico mapping of SNPs

SNP genotyping was performed using the Brassica 60 K Illumina SNP array, and SNP data were analyzed using Illumina GenomeStudio genotyping software. SNPs with call frequencies < 0.8 or MAF < 0.05 were excluded in the study. After processing, 31,846 SNP sequences were aligned with the genome sequences of *B. napus*, with an E-value cut-off of <‘1E-10’. The blast-hits with a minimum E value and a maximum score were selected for further analysis.

### Population structure, relative kinship and disequilibrium (LD) analysis

A subset of 4623 SNPs distributed evenly across the entire genome (missing data < 0.2, MAF > 0.2, and unique position on chromosome) was selected for population structure and relative kinship analysis (K). The model-based program STRUCTURE 2.3.4 software was used to estimate the population structure (Q) with a Bayesian Markov Chain Monte Carlo model (MCMC) [59]. Five independent runs were performed with a K-value (the putative number of genetic groups) from 1 to 10. Both the length of burning period and the number of MCMC replications after burning were set to 100,000 iterations under the “admixture model”. The optimal K value was determined by the log probability of the data [LnP(D)] in the STRUCTURE output and a statistic Δk based on the rate of change of LnP(D) between successive k values as described by Evanno et al. [29]. The results of replicate run from STRUCTURE were integrated to acquire a Q matrix with the CLUMPP software [60] and graphically displayed using DISTRUCT software [61]. The relative kinship matrix of the natural population was calculated using TASSEL 5.2.1 [62]. All negative values between two accessions were set to 0. The linkage disequilibrium (LD) between pair-wise SNPs (with MAF ≥ 0.05) on A- and C-subgenome was estimated by a parameter *r*^2^ calculated with the software TASSEL version 5.2.1 [62].

### Genome-wide association studies

Trait–SNP association analysis was separately performed in six models including Q (controlling for population structure), PCA (controlling for principal component), K (controlling for kinship), PCA + K (controlling for both principal component and kinship), Q + K (controlling for both population structure and kinship), and naïve (without controlling for population structure and kinship) model. The naive, Q and PCA models were performed using a general linear model (GLM); the K, Q + K and P + K models were performed using a mixed linear model (MLM). Both GLM and MLM were implemented in TASSEL 5.2.1 [62]. For each trait, the optimal model was selected based on the distribution of the -log_10_(*P*) values of each SNP against the expected value in a Quantile-quantile (Q-Q) plot [63]. A uniform threshold for the significant SNPs-trait association was set to −log_10_ (*P*) = 4.50 and a Manhattan plot was generated in the R package qqman [64]. Genes within ~ 250 kb upstream and downstream to the associated SNPs on A-subgenome and ~ 800 kb on C-subgenome were selected for identification of candidates.

### Transcriptome sequencing and identification of differentially expressed genes

Three high-wax load lines (HW) and three low-wax load lines (LW) were selected from the GWAS population for transcriptome sequencing, including Zhongshuang11, Shilijia, and Yangyou6 (HW) and SWU68, Tonglinghuaye, and Shengguang77 (LW). Leaf epidermis was collected from HW and LW lines, separately, with three biological replicates. In each replicate, two independent plants were sampled from each line and pooled together. Epidermal peels were manually dissected from leaves as a thin transparent film. Total RNA were extracted from peel samples for sequencing and quantitative reverse-transcription polymerase chain reaction (qRT-PCR). Sequencing library preparation and sequencing reactions were conducted at the Biomarker Technologies Corporation (Beijing, China). Sequencing libraries were constructed using NEBNext UltraTM RNA Library Prep Kit (NEB, USA). Subsequently, these libraries were sequenced on an Illumina platform and paired-end reads were generated.

Raw reads were transformed into clean reads after removing reads containing adapter, reads containing ploy-N and low-quality reads. These clean reads were then mapped to the *B. napus* reference genome sequence using Hisat2 tools soft. Quantification of gene expression levels were estimated by fragments per kilobase of transcript per million fragments mapped. Genes with significantly differential expression between HW and LW were identified based on the following criteria: false discovery rate (FDR) < 0.001 and absolute fold change ≥4. Transcription factor prediction was performed using BMKCloud (www.biocloud.net).

### GO and KEGG enrichment analysis of differentially expressed genes (DEGs)

To assess the biological significance of DEGs, GO enrichment analysis was implemented by the GOseq R packages based Wallenius non-central hyper-geometric distribution [65], which can adjust for gene length bias in DEGs. Statistically significant GO terms were obtained based on Kolmogorov-Smirnov like test. KEGG pathway enrichment analysis was performed by using the KOBAS software [66].

### Validation of DEGs by qRT-PCR

According to the results of combining GWAS with RNA-seq analysis, ten candidate genes were selected for qRT-PCR analysis. The primers for qRT-PCR are listed in Additional file 14: Table S7, and the *B. napus actin 7* gene (*BnACT7*) was used as the internal control. The total RNA was the same as used for RNA sequencing. The qRT-PCR assay was performed on a Bio-Rad CFX96 Real-Time PCR Detection System using the SYBR Premix Ex TaqII (Takara, Beijing, China). The relative gene expression levels were calculated using the 2^−ΔΔCt^ method. Three independent biological replicates, each with two technical replicates were analyzed for HW and LW, respectively.

## Supplementary information

**Additional file 1: Figure S1.** Histogram of wax traits investigated from 2016 to 2017.

**Additional file 2: Table S1.** Correlations among measured wax traits.

**Additional file 3: Table S2.** The information of 192 *Brassica napus* accessions and population structure used in this study.

**Additional file 4: Figure S2.** Quantile–quantile (QQ) plots from association analysis using six methods for 31 wax traits.

**Additional file 5: Figure S3.** Manhattan plots of GWAS results showing significant SNPs associated with 24 wax compounds in *Brassica napus* diversity panel.

**Additional file 6: Table S3.** Summary of SNPs significantly associated with wax traits.

**Additional file 7: Table S4.** Summary of RNA-Seq reads.

**Additional file 8: Figure S4.** The correlation between three biological replicates among high wax-load lines and low wax-load lines.

**Additional file 9: Figure S5.** Gene cluster that are differentially expressed in the *Brassica napus* epidermis with high wax coverage (HW) and low wax coverage (LW).

**Additional file 10: Table S5.** Differentially expressed transcription factor.

**Additional file 11: Figure S6.** GO categories of DEGs.

**Additional file 12: Figure S7.** Significantly overrepresented topGO terms of DEGs in the epidermis of *Brassica napus*.

**Additional file 13: Table S6.** Cuticular wax concentration.

**Additional file 14: Table S7.** Primers used for qRT-qPCR verification.

## Data Availability

The raw RNA-sequencing data from *B. napus* leaf epidermis were deposited in the NCBI under SRA accession number PRJNA602672 (https://www.ncbi.nlm.nih.gov/bioproject/PRJNA602672).

## Abbreviations

BLUP: Best linear unbiased prediction
DEGs: Differentially expressed genes
FAR: Fatty acyl-CoA reductase
FDR: False discovery rate
GWAS: Genome-wide association study
HW: High-wax load lines
LD: Linkage disequilibrium
LW: Low-wax load lines
MAH1: Midchain alkane hydroxylase 1
MLM: Mixed linear model
MLR: Multiple linear regression
qRT-PCR: Quantitative reverse transcription-PCR
SNPs: Single Nucleotide Polymorphisms
TFs: Transcription factors
WSD1: Wax ester synthase/acyl-CoA:diacylglycerol acyltransferase 1
