# Supplementary material for: A combination of genome-wide association study and transcriptome analysis in leaf epidermis identifies candidate genes involved in cuticular wax biosynthesis in Brassica napus
Source: BMC Plant Biol. 2020 Oct 6;20:458. doi: 10.1186/s12870-020-02675-y (PMC7541215; doi:10.1186/s12870-020-02675-y)
Supplement: Supplementary file 1 — Additional file 1: Figure S1. Histogram of wax traits investigated from 2016 to 2017. [file 12870_2020_2675_MOESM1_ESM.docx]

**Figure S1** Frequency distribution of wax contents investigated from 2016 to 2017. X-axis indicates the wax contents (μg·cm^−2^), while Y-axis indicates frequency distribution (%). Total C_29_, the sum of C_29_ Alkane, C_29_ Kettone and C_29_ 2-Alcohol; Alkane Pathway, the sum of products from alkane-forming pathway; 1-Alcohol Pathway, the sum of products from alcohol-forming pathway.
