# Supplementary material for: A combination of genome-wide association study and transcriptome analysis in leaf epidermis identifies candidate genes involved in cuticular wax biosynthesis in Brassica napus
Source: BMC Plant Biol. 2020 Oct 6;20:458. doi: 10.1186/s12870-020-02675-y (PMC7541215; doi:10.1186/s12870-020-02675-y)

**Figure S2** Quantile–quantile plots from association analysis using six methods for 31 wax traits. Distribution of P-values assuming associations (expected P-values) are represented as black lines; distribution of P-values calculated based on the six models (observed P-values) are represented as different colored lines. Total C_29_, the sum of C_29_ Alkane, C_29_ Ketone and C_29_ 2-Alcohol; Alkane Pathway, the sum of products from alkane-forming pathway; 1-Alcohol Pathway, the sum of products from alcohol-forming pathway.


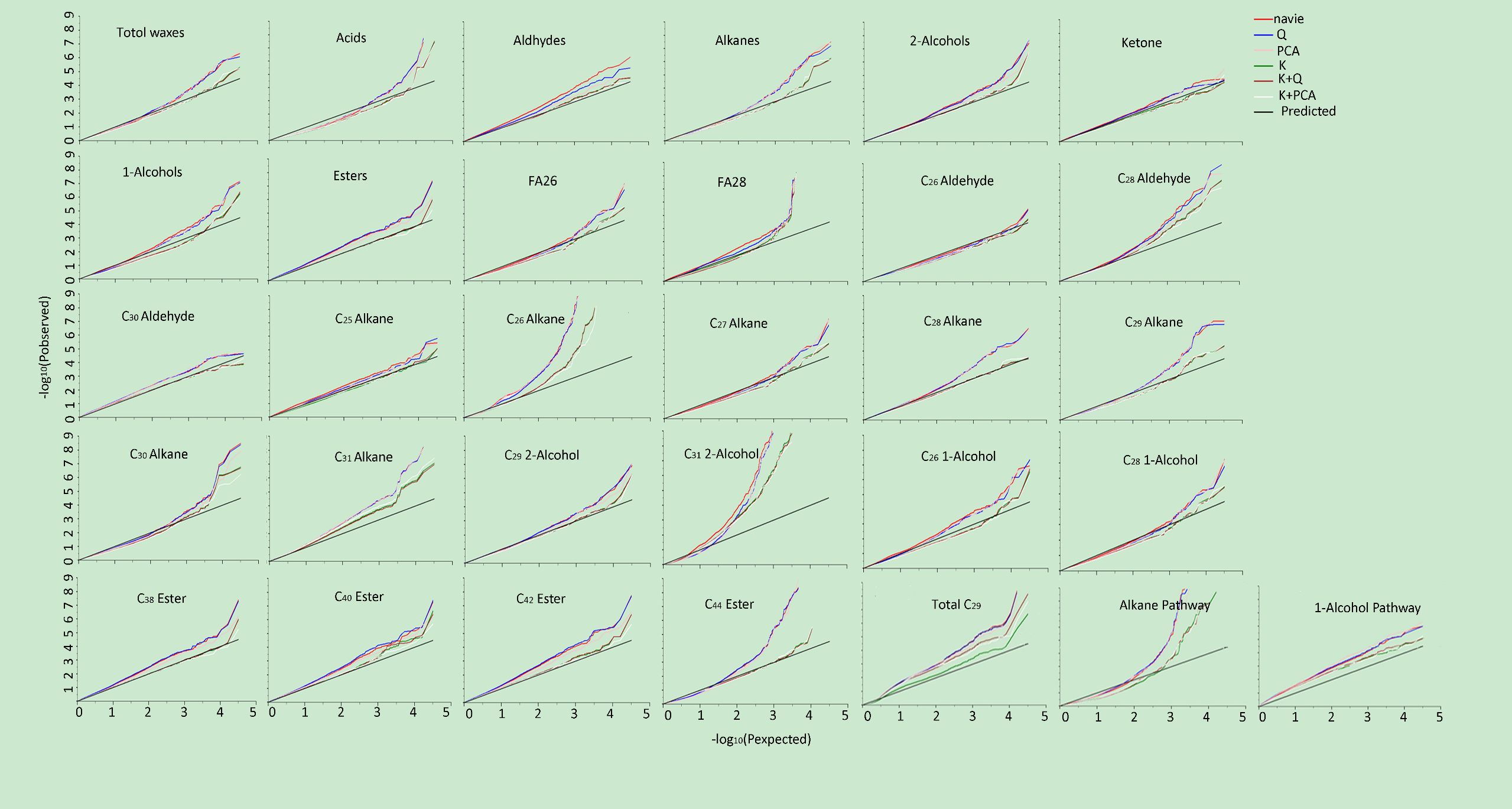

Supplement: Supplementary file 4 — Additional file 4: Figure S2. Quantile–quantile (QQ) plots from association analysis using six methods for 31 wax traits. [file 12870_2020_2675_MOESM4_ESM.docx]
