# Supplementary material for: A combination of genome-wide association study and transcriptome analysis in leaf epidermis identifies candidate genes involved in cuticular wax biosynthesis in Brassica napus
Source: BMC Plant Biol. 2020 Oct 6;20:458. doi: 10.1186/s12870-020-02675-y (PMC7541215; doi:10.1186/s12870-020-02675-y)

**Figure S3** Manhattan plots of GWAS results showing significant SNPs associated with 24 wax compounds in rapeseed diversity panel. X-axis shows the distribution of SNPs across 19 chromosomes while Y-axis shows Bonferroni corrections threshold. The black dashed horizontal line depicts the uniform significance threshold [–log_10_(p) = 4.5]. Total C_29_, the sum of C_29_ Alkane, C_29_ Ketone and C_29_ 2-Alcohol; Alkane Pathway, the sum of products from alkane-forming pathway; 1-Alcohol Pathway, the sum of products from alcohol-forming pathway.


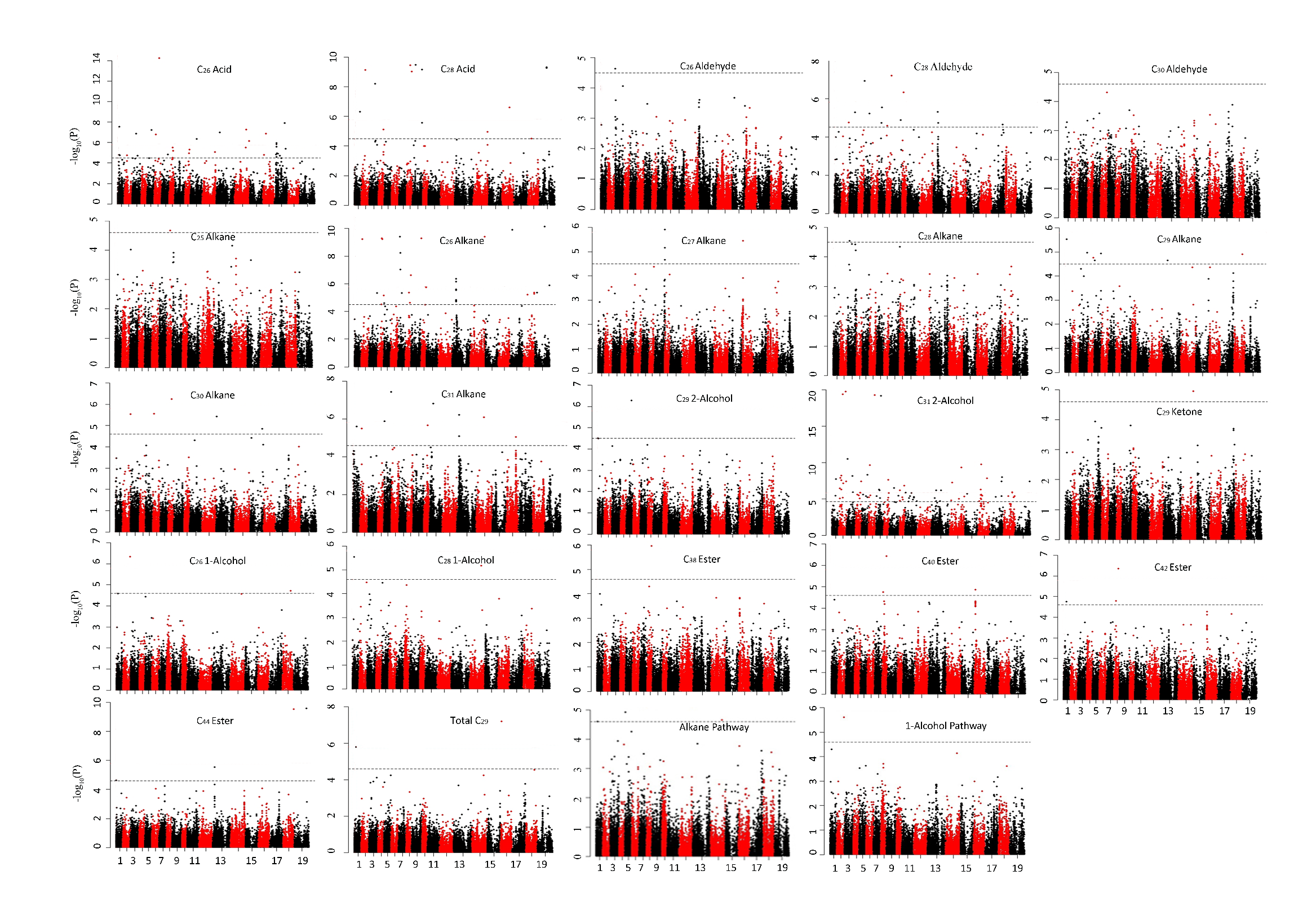

Supplement: Supplementary file 5 — Additional file 5: Figure S3. Manhattan plots of GWAS results showing significant SNPs associated with 24 wax compounds in Brassica napus diversity panel. [file 12870_2020_2675_MOESM5_ESM.docx]
