# Supplementary material for: A combination of genome-wide association study and transcriptome analysis in leaf epidermis identifies candidate genes involved in cuticular wax biosynthesis in Brassica napus
Source: BMC Plant Biol. 2020 Oct 6;20:458. doi: 10.1186/s12870-020-02675-y (PMC7541215; doi:10.1186/s12870-020-02675-y)

**Figure S5** Gene cluster that are differentially expressed in the *B. napus* epidermis with high wax coverage (HW) and low wax coverage (LW). Color index represents level of significance (P values). HW-1, HW-2, and HW-3 mix represent three biological replicates from high-wax load (HW) rapeseed lines, respectively, while LW-1, LW-2 and LW-3 mix represent replicates from low-wax load (LW) rapeseed lines.


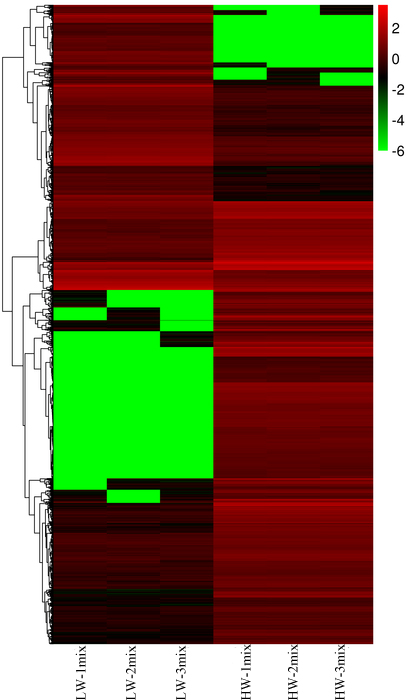

Supplement: Supplementary file 9 — Additional file 9: Figure S5. Gene cluster that are differentially expressed in the Brassica napus epidermis with high wax coverage (HW) and low wax coverage (LW). [file 12870_2020_2675_MOESM9_ESM.docx]
