# Supplementary material for: A combination of genome-wide association study and transcriptome analysis in leaf epidermis identifies candidate genes involved in cuticular wax biosynthesis in Brassica napus
Source: BMC Plant Biol. 2020 Oct 6;20:458. doi: 10.1186/s12870-020-02675-y (PMC7541215; doi:10.1186/s12870-020-02675-y)

**Figure S6** Histogram presentation of Gene Ontology classification. The results are summarized in three main categories, Biological process, Cellular component, and Molecular function. The left y-axis indicates the percentage of genes in a category, and the right y-axis indicates the number of genes in a category. “DE gene” indicates differentially expressed genes between high-wax load (HW) lines and low-wax load (LW) lines, while “All gene” indicates all expressed genes between HW and LW.


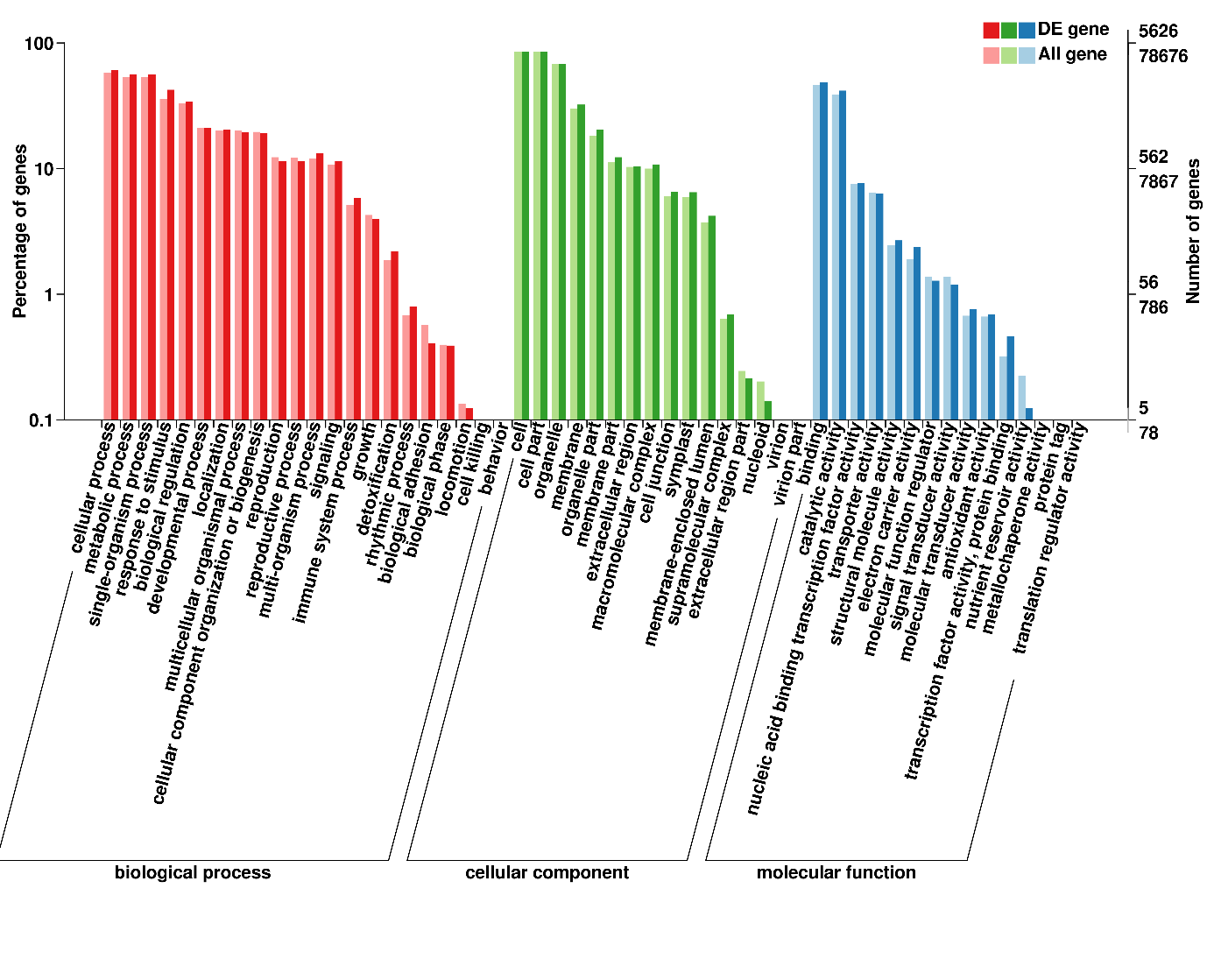

Supplement: Supplementary file 11 — Additional file 11: Figure S6. GO categories of DEGs. [file 12870_2020_2675_MOESM11_ESM.docx]
