# Supplementary material for: A combination of genome-wide association study and transcriptome analysis in leaf epidermis identifies candidate genes involved in cuticular wax biosynthesis in Brassica napus
Source: BMC Plant Biol. 2020 Oct 6;20:458. doi: 10.1186/s12870-020-02675-y (PMC7541215; doi:10.1186/s12870-020-02675-y)

**Figure S7** Significantly overrepresented topGO terms of DEGs in the epidermis of *B.napus*. “DEGs” indicates differentially expressed genes between high-wax load (HW) lines and low-wax load (LW) lines.


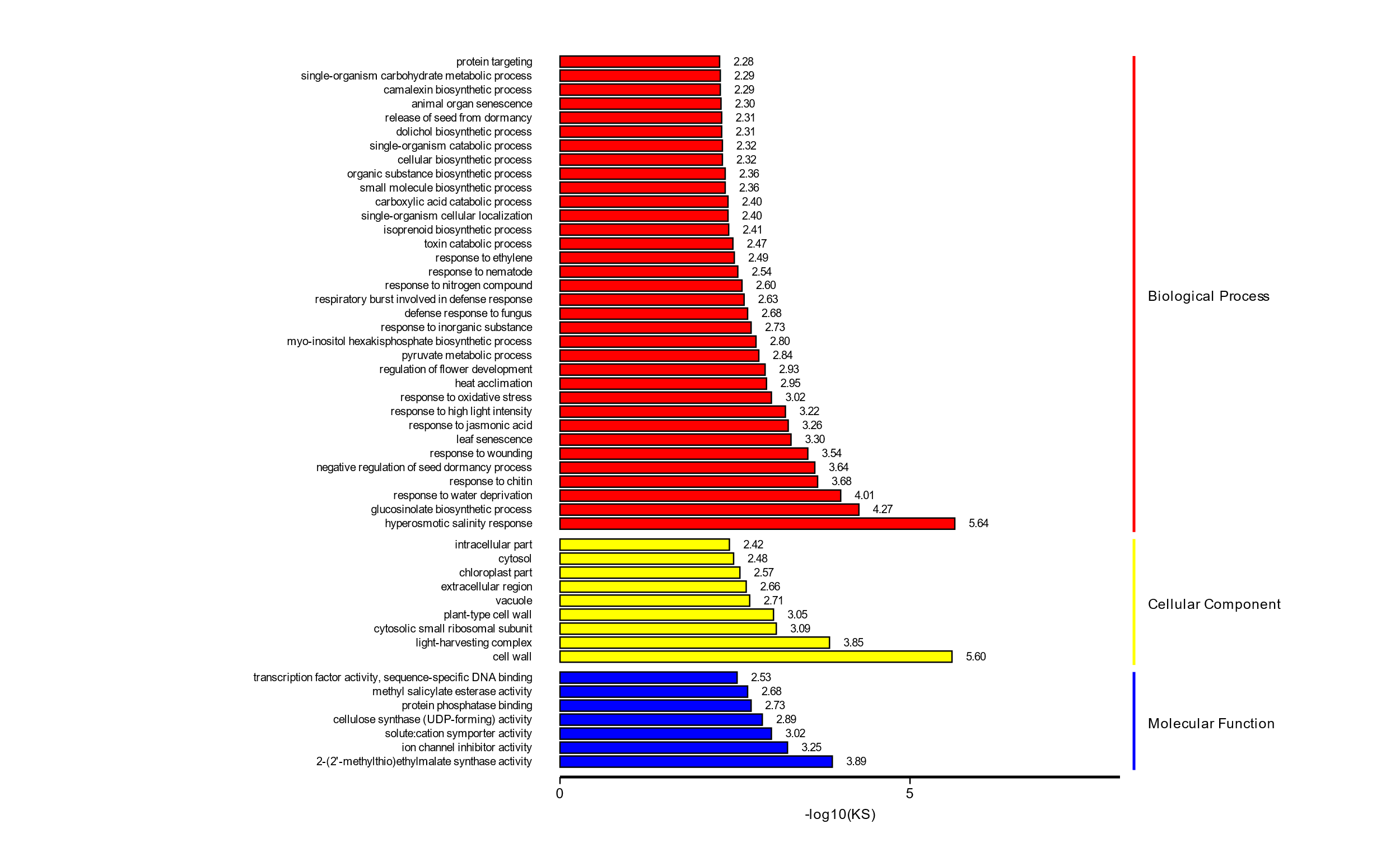

Supplement: Supplementary file 12 — Additional file 12: Figure S7. Significantly overrepresented topGO terms of DEGs in the epidermis of Brassica napus. [file 12870_2020_2675_MOESM12_ESM.docx]
